# Stator Dynamics Depending on Sodium Concentration in Sodium-Driven Bacterial Flagellar Motors

**DOI:** 10.3389/fmicb.2021.765739

**Published:** 2021-11-26

**Authors:** Tsai-Shun Lin, Seiji Kojima, Hajime Fukuoka, Akihiko Ishijima, Michio Homma, Chien-Jung Lo

**Affiliations:** ^1^Department of Physics and Center for Complex Systems, National Central University, Taoyuan City, Taiwan; ^2^Division of Biological Science, Graduate School of Science, Nagoya University, Nagoya, Japan; ^3^Graduate School of Frontier Biosciences, Osaka University, Suita, Japan

**Keywords:** bacterial flagellar motor, sodium-motive force, stator exchange, membrane protein, perfusion

## Abstract

Bacterial flagellar motor (BFM) is a large membrane-spanning molecular rotary machine for swimming motility. Torque is generated by the interaction between the rotor and multiple stator units powered by ion-motive force (IMF). The number of bound stator units is dynamically changed in response to the external load and the IMF. However, the detailed dynamics of stator unit exchange process remains unclear. Here, we directly measured the speed changes of sodium-driven chimeric BFMs under fast perfusion of different sodium concentration conditions using computer-controlled, high-throughput microfluidic devices. We found the sodium-driven chimeric BFMs maintained constant speed over a wide range of sodium concentrations by adjusting stator units in compensation to the sodium-motive force (SMF) changes. The BFM has the maximum number of stator units and is most stable at 5 mM sodium concentration rather than higher sodium concentration. Upon rapid exchange from high to low sodium concentration, the number of functional stator units shows a rapidly excessive reduction and then resurrection that is different from predictions of simple absorption model. This may imply the existence of a metastable hidden state of the stator unit during the sudden loss of sodium ions.

## Introduction

The cell membrane is not only the barrier for life but also the working place for many essential cellular functions. Membrane proteins show rich dynamics such as gating (Moreau et al., 2014), diffusing (Oswald et al., 2014; Lin et al., 2018), and exchange (Leake et al., 2006; Tusk et al., 2018; Armitage and Berry, 2020). Earlier investigations focused on the mechanical properties such as the gating mechanism or the diffusivity of membrane proteins. However, very little is known for the membrane protein energetic dynamics. In this report, we use the sodium-driven bacterial flagellar motor (BFM) as an example to study the energetic coupling dynamics of the protein complex with high-throughput and high-resolution optical measurements.

**FIGURE 1:**
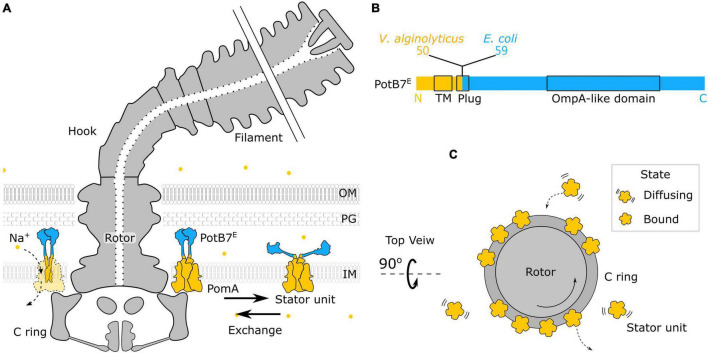
Structure of chimeric bacterial flagellar motor. **(A)** Schematic of the chimeric flagellar motor side view. A stator unit can diffuse on the membrane or bind to the motor. A bounded stator unit can interact with the C ring of the rotor and drive the rotor to rotate. The stator is composed of the PomA (orange) from *Vibrio alginolyticus* and the chimeric PotB7^E^. The PotB7^E^ joins the N terminus of *V. alginolyticus* PomA to the periplasmic C terminus of *Escherichia coli* MotB. The blue part is from *E. coli*, and the orange part is from *V. alginolyticus*. The chimeric stator units utilize the electrochemical energy of sodium ions (orange dots) to the motor rotation. **(B)** Schematics of the primary structure of a PotB7^E^. The black boxes represent the identified functional areas. OM, outer membrane; PG, peptidoglycan; IM, inner membrane; TM, transmembrane. **(C)** Top view of the flagellar motor. Stator units dynamically assemble to a motor (Bound) or disassemble from a motor and diffuse on the membrane pool (Diffusing).

The bacterial flagellum is a large molecular complex spanning across the membranes with extracellular flagellar filaments, universal joint (hook), and motor (rotor and stator) (Nirody et al., 2017; Nakamura and Minamino, 2019), as shown in Figure 1A. The rotation of the motor is powered by the transmembrane ion flux through the stator driven by the ion-motive force (IMF). The common driving ions are proton (H^+^) and sodium ions (Na^+^). About a dozen stator units are bound to the periphery of the motor to interact with the rotor independently (Figures 1A–C). A stator unit is composed of 5 MotA(PomA) and 2 MotB(PomB). A MotA(PomA) protein has four transmembrane segments, and the segment between the second and third transmembrane region is responsible for the interaction with the rotor. A MotB(PomB) protein has one transmembrane segment, a plug segment for ion flux control, and a large periplasmic segment with an OmpA-like domain for cell wall binding, as shown in Figure 1B. A model has been proposed in which the MotA(PomA) pentamer ring rotates with respect to the axis of MotB(PomB) dimer due to the influx of ions (Deme et al., 2020; Santiveri et al., 2020). The direct physical interaction between specific residues in FliG and PomA/MotA was demonstrated and has provided a mechanism with gear-like motion by the stator–rotor interaction in the flagellar motor (Terashima et al., 2021). The ion movement through the stator is driven by the IMF that comprises electrical and chemical potentials (Nirody et al., 2017).

Subunits of BFMs such as stator units have been found to be dynamic, dating back to the 1980s with so-called “resurrection” experiments (Block and Berg, 1984; Blair and Berg, 1988; Reid et al., 2006). In the stator genes deleted *Escherichia coli* strain, the stator proteins can be expressed from inducible plasmid, and the BFM rotation can be restored in a series of stepwise speed increments. Each speed increment step indicates that the addition of a functional stator unit contributed to the motor rotation. More recently, by using green fluorescent protein (GFP)–fused stator units and fluorescence recovery after photobleaching, the protein exchange of stator units in functional BFMs has been observed (Leake et al., 2006). The stator units can be unbound by reducing IMF, and the stator resurrection can be achieved by restoring the IMF (Tipping et al., 2013a; Sowa et al., 2014). Later it was found that the mechanical load can also affect the number of stator units (Lele et al., 2013; Tipping et al., 2013b; Chawla et al., 2017; Wadhwa et al., 2019). A catch-bond model, describing the bond between stator unit and motor strengthens with applied force (Nord et al., 2017a; Nirody et al., 2019), provides a new insight into the stator–rotor interaction. The number of functional stator unit is therefore not only depending on external load but also IMF and any other factors that would affect the force generation process. In other words, the rotation speed of a BFM depends on the external load, IMF, and the number of bound stator units.

In the stator unit recruitment model, there is a pool of unbound stator units diffusing on the membrane, with the plug of ion channel closed, as shown in Figure 1. It has been shown that the plug region of PomB prevents the ion influx by blocking the rotation of the rotor as a spanner to interact with the periplasmic loops of PomA (Homma et al., 2021). Once the stator unit is incorporated into the rotor, the interaction between FliG and stator units promotes stator–peptidoglycan binding, completing the assembly (Terahara et al., 2017; Kojima et al., 2018; Nord and Pedaci, 2020). A simple Hill–Langmuir absorption model has been proposed to describe the dynamic response of stator unit to the change of external load (Nord et al., 2017a; Wadhwa et al., 2019). The model assumes that each stator unit can be either diffusing freely on the membrane or binding to a motor. The unbinding rate *k*_off_ and the binding rate *k*_on_ describe the probability of a stator unit switching between the two modes and can be affected by the external load. When the external load changes, the number of bound stator units would adjust to the new state with an exponential transition. However, the detailed role of coupling ions to the stator unit assembly and disassembly dynamics remains unclear.

The chimeric stator, PomA/PotB7^E^, was developed in 2003 initially for the research of ion selectivity (Asai et al., 2003). The PotB7^E^ joining the N terminus of *Vibrio alginolyticus* PomA to the periplasmic C terminus of *E. coli* MotB can function with native PomA from *V. alginolyticus* as the sodium stator in Δ*motAmotB E. coli* cells, shown in Figures 1A,B. The sodium-driven stator in *E. coli* provides a useful experimental system to manipulate the sodium gradient of SMF without interfering membrane potential (Lo et al., 2007). The SMF and the torque–speed relationship of chimeric BFM has been reported (Lo et al., 2006, 2007, 2013; Inoue et al., 2008; Nord et al., 2017b). However, very little is known for the critical ion concentration-dependent stator dynamics. Here, we use microfluidic devices to perform computer-controlled fast perfusion and high-throughput experiments to investigate the stator dynamics to the sodium ion concentrations. We found the chimeric BFMs maintain constant speed over a wide range of sodium concentrations by adjusting stator units in compensation to the SMF changes. The number of stator units shows a reduction and then resurrection during a step-down sodium transition. This may imply the existence of a metastable hidden state of the stator unit during the sudden loss of sodium ions.

## Materials and Methods

### Bacterial Strains and Culture Conditions

Bacterial strains and growth medium used in this study are listed in Table 1. Briefly, the chimera strain (YS34 with pYS11 and pYS13) with sodium-type stator units in *E. coli* was used (Sowa et al., 2005; Lo et al., 2006, 2007). The wild-type strain with proton-type stator units (SYC12) was modified from *E. coli* strain RP437 by replacing *fliC* on the genome to the sticky filament *fliC^st^* (Scharf et al., 1998; Ryu et al., 2000; Hata et al., 2020). A dual fluorescent protein fused strain (fluorescent chimera) with chimeric sodium-type stator units in *E. coli* was constructed and derived from JHC36 (Inoue et al., 2008). The stator-unit protein PomA is fused with enhanced GFP (eGFP), and the rotor protein FliN is fused with mCherry.

**TABLE 1:** The list of bacterial strains and medium used in this study.

	Description	References
**Bacterial strains**		
YS34	*fliC*::Tn10, △*pilA, △motAmotB, △cheY*, RP4979 derivative	Sowa et al., 2005
Chimera	YS34 + pYS11 + pYS13	Sowa et al., 2005
SYC12 (wild type)	*fliC*::*fliC^st^*, otherwise wild type, RP437 derivative	Hata et al., 2020
EFS023	*fliC*::*fliC^st^*, △*motAB, △fliN*, JHC36 derivative	This study
Fluorescent-chimera	EFS023 + pTSK121 + pTSK108	This study

**Plasmids**		
pYS11	*fliC* sticky filaments, ampicillin resistance, pBR322 derivative	Sowa et al., 2005
pYS13	*pomA*/*potB7*^E^, IPTG inducible, chloramphenicol resistance, pMMB206 derivative	Sowa et al., 2005
pTSK121	*egfp-pomA/potB7^E^*, arabinose inducible, ampicillin resistance, pBAD24 derivative.	This study
pTSK108	*mCherry-fliN*, salicylate inducible, chloramphenicol resistance, pKG116 derivative.	This study

**Growth medium**		
LB	1% tryptone (BD Bacto), 0.5% yeast extract (cat. no. Y1625, SIGMA), 0.5% NaCl	
TB	1% tryptone (BD Bacto), 0.5% NaCl	

Cells from frozen stocks were cultured in 2 mL of Luria–Bertani broth (LB) overnight at 37°C. Then, overnight culture was diluted 40× to 2 mL in tryptone broth (TB) for 5 h at 30°C. The required antibiotics and inducers were added to the growth medium to preserve the plasmids and express the proteins. Antibiotics concentrations were 34 μg/mL for chloramphenicol and 50 μg/mL for ampicillin. The inducer concentrations were 25 μM for isopropyl-β-D-thiogalactoside, 0.002% for arabinose, and 313 nM for sodium salicylate.

### Beads Assay and Sample Preparation

To truncate flagellar filaments, 1.2 mL of cells in TB was sheared by passing 30 times back and forth through a shearer, a custom-made device with two syringes mounted to two 26-gauge needles connected by a tube. The sheared cells were washed three times and concentrated to OD 1.0 through centrifugation (5,200*g*, 2 min) with motility buffer (MB, 10 mM potassium phosphate, *X* mM NaCl, *Y* mM KCl, 0.1 mM EDTA, pH 7.0), where *X* depends on the designed sodium ion concentration, and the total ionic strength (*X* + *Y*) is fixed to 85 mM.

**FIGURE 2:**
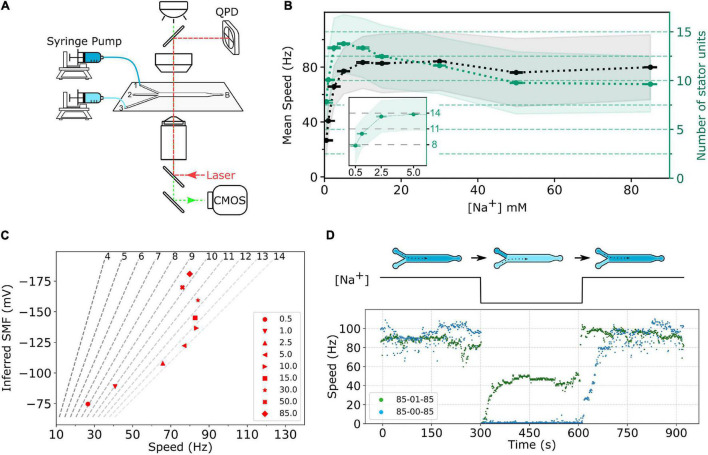
**(A)** Schematic of the experimental setup. The bacterial cells are immobilized on the surface of a three-way microfluidic chamber. The BFMs’ rotations were recorded by a high-speed CMOS camera (green light path), and one BFM rotation can be measured simultaneously by a BFI system (red light path). Two syringe pumps connect to inlet ports 1 and 3, respectively, to supply two different sodium concentrations of MB. Slow constant flow (20 μL/min) is maintained all the time to remove any bacterial waste. **(B)** Sodium ion dependency of BFM speed. The black dots, gray shaded area, and error bar represent average BFM speed, the standard deviation, and the standard error of the mean, respectively, under the load of 1-μm beads as [Na^+^]_ex_ varies. The green dots, light-green shaded area, and error bar show the inferred number of stator units, standard deviation, and standard error of the mean, respectively. Inset: Enlarge the number of stator unit data in the low-sodium region. The numbers of measured BFMs are *N* = 805, 2,463, 1,489, 1,785, 1,149, 1,929, 7,047, 2,562, and 6,833 for [Na^+^]_ex_ = 0.5, 1, 2.5, 5.0, 10.0, 15.0, 30.0, 50.0, and 85.0 mM, respectively. **(C)** BFM states diagram. The speed of BFM is dependent on the SMF and the number of stator units. The red dots are the BFM average speed in panel **(B)** and the SMF at that [Na^+^]_ex_. The dashed lines are stator unit number contour line from Eq. 2 labeled at the top. **(D)** BFM speed response to the perfusion experiments. The top diagram shows the experimental course with *X*-*Y*-*X* mM [Na^+^]_ex_ perfusion. There are three stages in each experiment, and each stage remains approximately 5 min. The numbers in the legend indicate the [Na^+^]_ex_ at each stage. The bottom figure shows two represented experimental BFI data sets. For the 85-00-85 mM experiment (blue dots), the BFM speed drops to zero as [Na^+^]_ex_ was depleted and resurrected by stepwise speed increase as [Na^+^]_ex_ restored to 85 mM. For 85-01-85 mM experiments (green dots), the BFM speed drops to zero and then resurrected to a steady speed as [Na^+^]_ex_ changes to 1 mM. The speed returned back to high speed level as [Na^+^]_ex_ restored to 85 mM.

To perform fast perfusion experiments, we used 3-way microfluidic chambers (μ-Slide III 3in1, cat. no. 80311, ibidi). The channel slide was first coated with 60 μL of 0.001 % (wt/vol) poly-L-lysine for 1 min from port B, shown in Figure 2A. Then, the coating solution was removed by adding 180 μL MB to each port—1, 2, 3—and the waste was withdrawn from port B. The chamber slide was set up to a microscope, and ports 1 and 3 of the slide were respectively connected to two programmable syringe pumps (Fusion 400, Chemyx) loaded with MB containing specific sodium concentration.

The cells were injected into the slide from port 2 and waited for 30 min for the adhesion. The unattached cells were flushed away by MB from port 2. After that, 0.125 % (wt/vol) of polystyrene beads (0.99-μm diameter, cat. no. 07310, PolySciences) was injected into the slide from port 2 and waited for 30 min for beads attaching to flagella. The unattached beads were flushed away with the flow of MB from port 2. To remove all potential residues, the extra 1 mL MB perfusion from ports 3 and 1 was performed at a flow rate 2,000 μL/min. Finally, a slow washing flow of 20 μL/min was maintained in the channel.

The high-throughput BFM rotation experiments were imaged by a Nikon Ti-U microscope equipped with a 100× objective (N.A. 1.49), a 0.6× relay, and a CMOS camera (UI-3370CP-M-GL, IDS) recording at 450 fps with 2,048 × 350 pixel^2^. Simultaneous high-spatial-resolution BFM rotation recording of one BFM can be achieved by back focal-plan interferometry (BFI), shown in Figure 2A. The fluorescence experiments were conducted on the same microscope with laser illumination and imaged by an EMCCD camera (Evolve 512, Photometrics). All experiments were conducted at 25°C ± 1°C.

### Perfusion Experiment and Data Collection

All perfusion and recording were controlled by the computer for consistency. The high-throughput BFM rotation images were collected from 80 positions in the middle of the channel slide at 450 fps and 1 s long each. During the recording, the channel was kept at a slow constant flow (20 μL/min) of fresh MB.

In sodium switch experiments, three-stage sodium ion concentration sequences were applied to the channel indicated as *X*-*Y*-*X* sequence, where each number represents the extracellular sodium ion concentration ([Na^+^]_ex_) in mM. We used the camera program (Streampix 7) and Labview to control perfusion and imaging timing.

The experimental sequence is as follows. First, the cells were maintained at *X* mM [Na^+^]_ex_ by the pump connected to port 1 with a constant flow (20 μL/min) for 5 min. The pump was stopped, and the second pump connected to port 3 was activated to perfuse 75 μL with a fast flow rate (2,000 μL/min) for approximately 3 s to switch the [Na^+^]_ex_ to *Y* mM. Then, the flow rate was turned down to a slow constant flow (20 μL/min). After 5 min, the pump switching process was repeated, but the order of pumps was inversed to restore the [Na^+^]_ex_ to *X* mM for another 5 min.

The image data were collected with dynamic intervals where higher-resolution recording is applied to the fast speed transition regions, Supplementary Figure 1. For the first stage, approximately 5-minute period (Δt_1_), the steady rotations were recorded 1 s in every 20-second durations (duty cycle 1:20). For the second stage, the recording sequence was continuous 7 s (Δ*t*_2–1_, duty cycle 1:1), 1 s in every 3 s for a total of 76 s (Δt_2–2_, duty cycle 1:3), and 1 s in every 20 s (Δt_2–3_, duty cycle 1:20) for the rest 221 s. Stage 3 is a repeat of stage 2. One BFM rotation was recorded continuously with high spatial and temporal resolution by BFI in the whole experiments at 1-kHz sampling rate (Figure 2A).

### Data Analysis

To image data, the rotation speeds of BFMs were monitored by the attached 1-μm beads. The stage drift was corrected prior to further processing. The beads’ center positions were found by weighting of the intensity signal. For BFI data, the beads’ positions were derived by the quadrant photodiode signal as in previous studies (Rowe et al., 2003; Sowa et al., 2005). During the fast flow, the images of attached beads were affected approximately 5 s, including the relaxation of the flow switch. The speed data were excluded in these periods.

The speed was calculated from the power spectrum of combined *x*-*y* position data (Rowe et al., 2003) of 1 s. To ensure the data quality from the bead assay, three criteria were applied to the data analysis. First, the radical fluctuation is small, and the orbit is steady. Second, the rotation radius is within 180 and 220 nm. Third, the rotation speed is stable without any sudden stop unless for low [Na^+^]_ex_ conditions.

To calculate the sodium-motive force (SMF), Eqs. 1, 3, and 4 and membrane potential (*V*_m_) from the previous report (Lo et al., 2007) were used. The condition [Na^+^]_ex_ = 0 leads to an undefined value of Eq. 3. We estimated the maximum sodium residue is 0.08 mM in the sodium-free MB from the ingredients’ impurities. Therefore, we use [Na^+^]_ex_ = 0.08 mM for the SMF estimation in the condition [Na^+^]_ex_ = 0.

## Results

### Sodium-Dependent Stator Assembly at Equilibrium

First, we investigated the BFM speed at a different [Na^+^]_ex_. The cells are in the desired [Na^+^]_ex_ at least for 1 h. Similar to the sodium-driven BFM in *V. alginolyticus* (Sowa et al., 2003), the speed of sodium-driven chimeric BFM in *E. coli* is sodium-dependent with a saturation speed of approximately 80 Hz at 10 mM [Na^+^]_ex_ with 1-μm bead load, as shown in Figure 2B.

At 1-μm bead high-load condition, the rotation speed is proportional to the number of stator units (Inoue et al., 2008). Here, we assume the speed contribution from each stator unit is equal and independent of the rest of bound stator units. *E. coli* has partial homeostasis of internal sodium concentration,


(1) $${\left[{\text{Na}}^{+}\right]}_{\text{in}}=7.2\,{\left({\left[{\text{Na}}^{+}\right]}_{\text{ex}}\right)}^{0.2},$$

where [Na^+^]_in_ and [Na^+^]_ex_ are the intracellular and extracellular sodium ion concentrations, respectively (Lo et al., 2006). Furthermore, the relation between the sodium ion gradient and the single stator unit speed has been reported (Lo et al., 2006),


(2) $$F_{s}=6.408-2.745\,\log\,\frac{{\left[{\mathrm{Na}}^{+}\right]}_{\mathrm{in}}}{{\left[{\mathrm{Na}}^{+}\right]}_{\mathrm{ex}}}$$

where *F*_s_ (Hz/stator) is the rotational speed contributed from a single stator unit. Using these relations, the rotational speed contributed by a single stator unit (*F*_s_) can be obtained.

The number of stator units in the steady state is calculated by dividing the average speed by *F*_s_, as shown in Figure 2B. The number of stator units is not a monotonic increase with the increase in [Na^+^]_ex_. The stator unit number increases rapidly from 0 to 2.5 mM [Na^+^]_ex_ and reaches its maximum at approximately 5 mM [Na^+^]_ex_ (Figure 2B, inset). From 10 to 85 mM [Na^+^]_ex_, the number of stator units reduces from 14 to approximately 10. For 85 mM [Na^+^]_ex_, the stator unit number is consistent with the previous report (Reid et al., 2006). To further confirm this new finding that the number of bound stator units around the motor does not increase with the increasing [Na^+^]_ex_, we measured the average fluorescent intensity of functional BFMs in eGFP-PomA strain, Supplementary Figure 2. The results confirmed that there are more stator units in the 1 and 5 mM [Na^+^]_ex_ conditions than in 85 mM [Na^+^]_ex_. This is different from the general belief that the stator is more stable in the higher [Na^+^]_ex_.

### Stator Number Under Dynamic Sodium-Motive Force

The *V*_m_ remains constant in these experimental conditions (Lo et al., 2007). Therefore, the SMF,


(3) $$\text{SMF}=V_{\text{m}}-2.3\,\frac{k\,T}{e}\,\log\,\frac{{\left[{\mathrm{Na}}^{+}\right]}_{\mathrm{ex}}}{{\left[{\mathrm{Na}}^{+}\right]}_{\mathrm{in}}}$$

can be calculated, where *k* is the Boltzmann’s constant, *T* is the absolute temperature, and *e* is the elementary charge. To illustrate the status of BFM, we plot the BFM state diagram as the function of average speed and SMF with stator-unit number contour lines (Figure 2C). It is clear that the BFM remains constant speed over a wide range of [Na^+^]_ex_, and the number of stator units is maximized at approximately 5 mM [Na^+^]_ex_.

To further investigate the BFM stator units dynamics, we designed fast perfusion experiments for BFM to switch from different states of [Na^+^]_ex_. Figure 2D shows the typical time course for two experiments with step-down and step-up of [Na^+^]_ex_. For the 85-00-85 mM sequence, the BFM speed is initially at approximately 80 Hz in 85 mM [Na^+^]_ex_ and stopped immediately as 0 mM [Na^+^]_ex_ introduced. The rotational speed recovered in a stepwise fashion within few minutes after the [Na^+^]_ex_ is restored to 85 mM (Figure 2D, blue dots) as reported (Sowa et al., 2014). For 85-01-85 mM experimental sequence, from the steady state BFM speed and stator unit number data (Figure 2B), we expect there is a speed change, but no stator unit number changes. However, the BFM speed shows two-stage speed changes with an overshooting and then recovery to the steady-state speed during the step-down [Na^+^]_ex_. When the [Na^+^]_ex_ is restored to the initial 85 mM level, the BFMs speed up to the original level quickly.

Because the BFM speed depends on both SMF and the number of stator unit, it is necessary to analyze the SMF contribution during the perfusion. *E. coli* has partial homeostasis of internal sodium concentration and response time for the physiological adjustment to the new steady intracellular sodium concentration (Lo et al., 2006). The dynamic response of intracellular sodium ion concentration can be described as follows:


(4) $${\left[{\mathrm{Na}}^{+}\right]}_{i\,n}^{t}={\left[{\mathrm{Na}}^{+}\right]}_{i\,n}^{i}+\left({\left[{\mathrm{Na}}^{+}\right]}_{i\,n}^{f}-{\left[{\mathrm{Na}}^{+}\right]}_{i\,n}^{i}\right)\,\left(1-e^{-t\,/\,\tau }\right)$$

where ${\left[{\mathrm{Na}}^{+}\right]}_{i\,n}^{i}$ and ${\left[{\mathrm{Na}}^{+}\right]}_{i\,n}^{f}$ are the stationary intracellular sodium concentration before and after perfusion. The response time constant τ is 29 s as reported (Lo et al., 2006). Given initial and final [Na^+^]_ex_, the ${\left[{\mathrm{Na}}^{+}\right]}_{i\,n}^{i}$ and ${\left[{\mathrm{Na}}^{+}\right]}_{i\,n}^{f}$ can be calculated by homeostasis relation (Eq. 1). Then, we are able to calculate the sodium gradient and SMF as the functions of time from Eqs. 3, 4 and estimate the single stator speed by Eq. 2.

**FIGURE 3:**
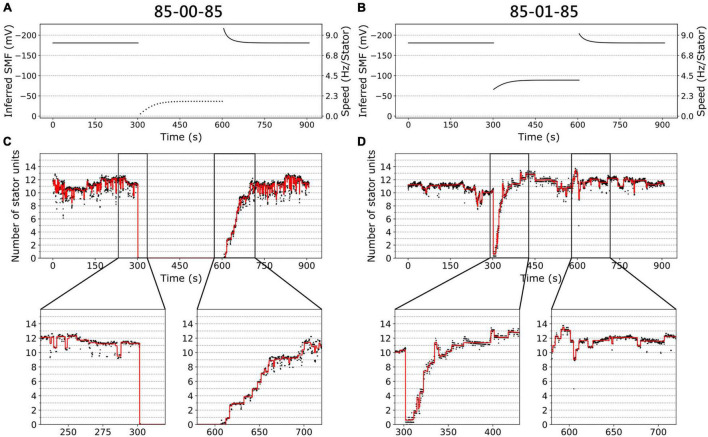
BFM speed and stator dynamics. **(A)** The inferred SMF as a function of time for experiment 85-00-85 mM sequence. The dotted line represents an estimated SMF with the assumption of nonzero [Na^+^]_ex_ = 0.08 mM because the sodium gradient term is undefined at zero sodium ion concentration. The right-hand-side *y* axis shows the speed per stator unit. **(B)** The inferred SMF as a function of time for experiment 85-01-85 mM sequence. **(C)** The stator unit number of a representative trace in an experiment with 85-00-85 mM sequence, same data in Figure 1D. The insets are the magnification of transition courses (rectangle region). The red line is the stator number filtered by the edge-preserving Chung–Kennedy filter (Chung and Kennedy, 1991) and was stepped by Student *t* test (Leake et al., 2004). **(D)** The stator unit number of a representative trace in experiment 85-01-85 mM sequence, same data in Figure 1D.

Figures 3A,B show the inferred SMF results for the two conditions of the same traces in Figure 2D. The SMF would decrease (more positive) while switching from high to low [Na^+^]_ex_. As a result, the speed per stator unit also decreases in the lower [Na^+^]_ex_.

We applied the above method to estimate the number of stator units in the whole perfusion experiments (Figures 3C,D). For 85-00-85 mM perfusion experiments, the speed recovery trace (0–85 mM) shows stepwise but not equal increment speed recovery (Figure 2D, blue dots). This is because the SMF adjusted as well. By our estimation, considering the SMF dynamics, the stepwise changes of stator unit match the discrete stator unit number, which suggests our estimation is reasonable, as shown in Figure 3C.

For 85-01-85 mM perfusion experiment, although the steady-state speed changes, however, the stator unit number is close to 11 in both 1 and 85 mM [Na^+^]_ex_ conditions (Figure 3D). Therefore, we expect there is a direct speed change without number of stator unit changes. Surprisingly, during the 85-01 mM perfusion, the number of stator units reduced to zero and then recovered to the same level as 85 mM (Figure 3D). This is very different from the simple Hill–Langmuir absorption model of stator unit dynamics. Further investigation is presented in section “Stator Response to Sodium Concentration Shift.”

### Sodium-Dependent Stator Kinetics

To quantify the sodium ion dependency of stator units, we systematically investigate the BFM speed and stator unit number dynamics between various [Na^+^]_ex_. The experiments were designed as a three-stage perfusion sequence of *X*-*Y*-*X* mM [Na^+^]_ex._

**FIGURE 4:**
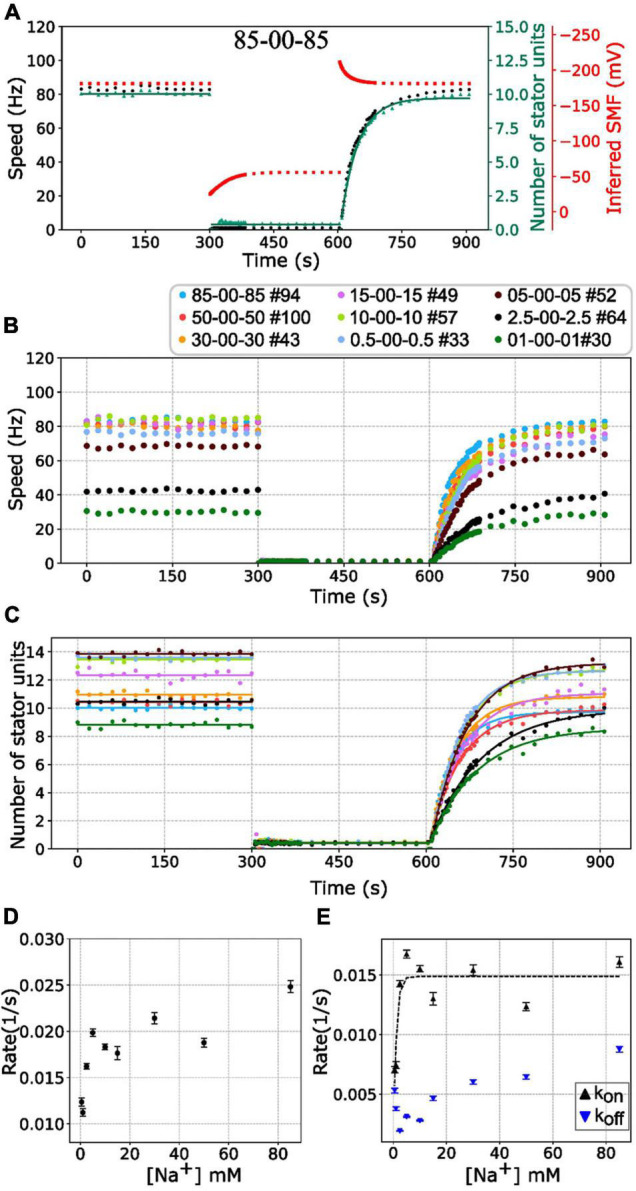
BFM speed and stator number kinetics to zero [Na^+^]_ex_. **(A)** Perfusion experiment of 85-00-85 mM sequence. The black dots show the average BFM speed. The red dots represent the inferred SMF. The green dots and line represent the number of stator units and fitting result to the simple Hill–Langmuir absorption model **(B)** BFM speed data collection of perfusion experiments with different [Na^+^]_ex_. The legend indicates the perfusion [Na^+^]_ex_ sequence and the number of BFM speed data collected. **(C)** The dots and lines show the number of stator units inferred from the speed results in B and the fitting results, respectively. **(D)** The recovery rate constants in the third stages (zero to high [Na^+^]_ex_) in C versus [Na^+^]_ex_. **(E)** The *K*_on_ and *K*_off_ according to the sodium concentration derived from panels **(C,D)**. The black tendency line for the *K*_on_ is obtained by fitting a simple exponential curve. The error bar in panels **(D,E)** represents the standard deviation of the fitting parameter.

First, we investigated the BFMs from different [Na^+^]_ex_ to the extreme condition with zero sodium. The BFM stator kinetics shows similar patterns in all conditions. The BFM is driven by sodium ion and stopped at zero [Na^+^]_ex_ immediately (Figures 4A,B). The BFM speed recovered from zero to its original level once the [Na^+^]_ex_ was restored in a few minutes (Figures 4A,B and Supplementary Figure 3). The stator unit numbers were then recovered as well (Figure 4C).

The simplest stator dynamics model has been reported and described by a reversible Hill–Langmuir model (Nord et al., 2017a; Wadhwa et al., 2019). This model assumes that there is a pool of stator units diffusing independently on the membrane. A rotor with *N*_max_ independent bound stator units was fixed on the membrane. A bound stator unit can unbind with a rate constant *k*_off_, and a free stator unit can bind to an empty site with a rate constant *k*_on_. To the average stator occupancy, *N(t)* follows:


(5) $$\frac{\text{dN}}{\text{dt}}=k_{\text{on}}\,\left(N_{\text{max}}-N\right)-k_{\text{off}}\,N$$

At steady state, that is, *dN/dt = 0*, the steady state stator occupancy is determined by *N*_s_ = *N*_max_/(1 + *K*_*D*_) where *K*_D_ = *k*_off_*/k_*on*_*.

When the motor encounters a state transition, the simple model predicts an exponential transition toward the new steady-state occupancy (Nord et al., 2017a; Wadhwa et al., 2019),


(6) $$N\,\left(t\right)=N^{\text{f}}+\left(N^{\text{i}}-N^{\text{f}}\right)\,e^{-K\,t}$$

where *N^f^* and *N^i^* are the number of stator units in the initial and final steady states, and


(7) $$k_{\text{on}}=\frac{N_{s}\cdot K}{N_{\text{max}}}\,;\,k_{\text{off}}=\frac{\left(N_{\text{max}}-N_{\text{s}}\right)\cdot K}{N_{\text{max}}}$$

The single exponential equation described by a constant rate *K* relates to the stators’ on/off rate as *K* = *k*_on_ + *k*_off_.

Based on this model, the stator resurrection process can be fitted by Eq. 6 (Figure 4C, line), and rate constant *K*, *k*_on_, and *k*_off_ can be obtained (Figures 4D,E). The recovery rate constant *K* increases as the [Na^+^]_ex_ increases (Figure 4D). In Figure 4E, the *k*_on_ is a constant in the range of [Na^+^]_ex_ = 5 to 85 mM, whereas the *k*_off_ increases in this range. At 5 mM [Na^+^]_ex_ conditions, there are more stator units because the *k*_off_ is at the lowest point. However, in the 2.5–0.5 mM [Na^+^]_ex_ range, *k*_on_ drops rapidly, whereas *k*_off_ increases a little.

Current understanding of stator unit assembly process comprises the first step of rotor–stator interaction and the second step of stator binding to the cell wall (Kojima et al., 2011; Kojima, 2015). The previous result showed the sodium ion can bind to stator units after its conformational change which leads to the plug open and the structural changes of the cytoplasmic loop (Onoue et al., 2019). That is an assembly-coupled stator activation. It has been reported that the assembly of stator units in the sodium-driven BFMs is sodium-dependent (Fukuoka et al., 2009). Our results support that at least 5 mM [Na^+^]_ex_ is required for stator units stabilization and interaction with the rotor.

However, the *k*_off_ raises as more sodium was introduced. The manner is similar to the load-dependent stator stability that the stator is less stable and *k*_off_ is higher in the low load conditions. It is the increment of the *k*_off_ that leads to the drop of stator unit in a higher sodium concentration. It might be interesting to test the stability of stator units in the extremely high sodium concentration within the cell physiology conditions.

### Stator Response to Sodium Concentration Shift

**FIGURE 5:**
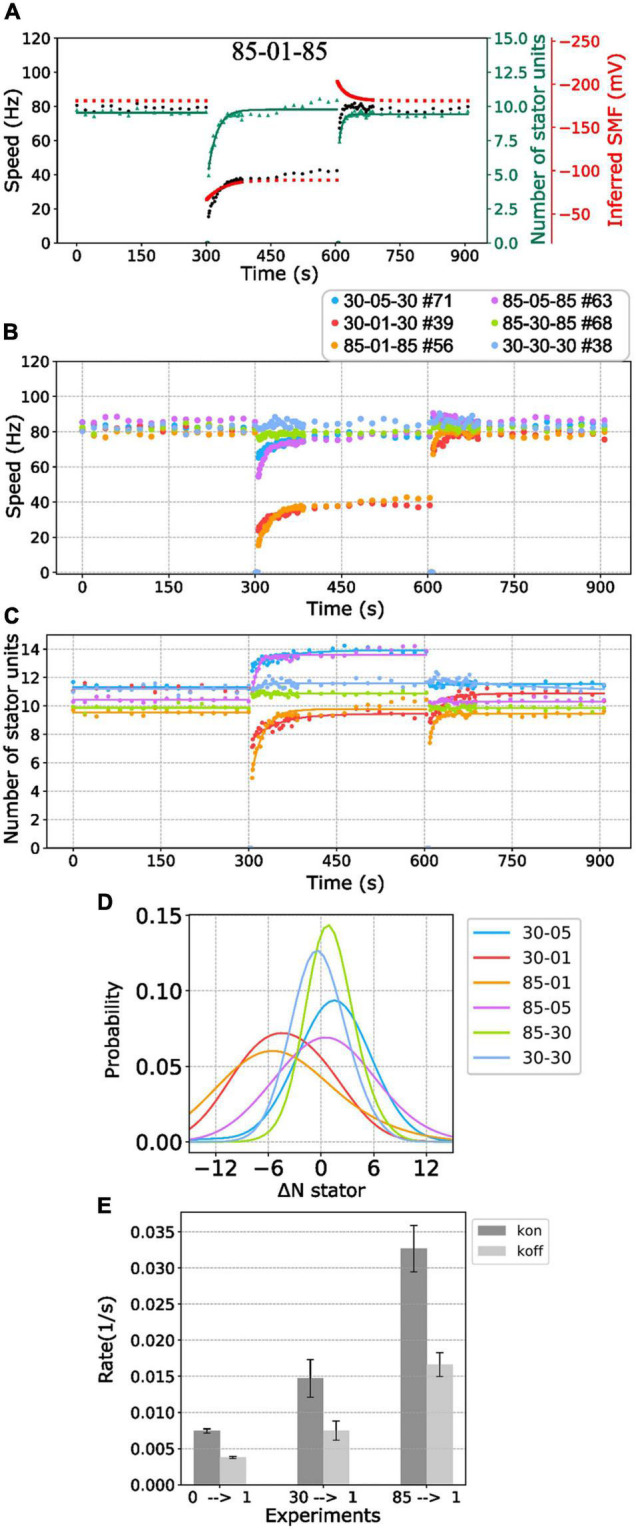
BFM speed and stator number kinetics to nonzero [Na^+^]_ex_. **(A)** Perfusion experiment of 85-01-85 mM sequence. The black dots show the average BFM speed. The red dots represent the inferred SMF. The green dots and line represent the number of stator units and its fitting result to the simple Hill–Langmuir absorption model. **(B)** BFM speed data collection of perfusion experiments from high to different nonzero different [Na^+^]_ex_. The legend indicates the perfusion [Na^+^]_ex_ sequence and the number of BFM speed data collected. **(C)** The dots and lines show the number of stator units inferred from the speed results in B and the fitting results, respectively. **(D)** Kernel density estimate of the number of stator unit differences between step-down sodium perfusion. **(E)** The *K*_on_*/K*_off_ for 0-1, 30-1, and 85-01 mM stator unit resurrection fitting results in C. The error bar is the standard deviation of the fitting parameter.

The BFM has mechanosensing capability for cells to adapt to the chemical and mechanical changes of environments (Nord et al., 2017a; Wadhwa et al., 2019). However, very little is known for the effect of driving ion concentration on the BFM stator dynamics. In the following section, we reported the investigation of stator adjustment to the nonzero [Na^+^]_ex_ change. Figure 5A shows the BFM speed under perfusion experiments of 85-01-85 mM [Na^+^]_ex_. BFMs rotate stably at 80 Hz in the 85 mM [Na^+^]_ex_. There was a dramatic speed drop when 1 mM [Na^+^]_ex_ was applied and then resurrected to the 40-Hz steady-state speed. And then, a speed increased when the 85 mM [Na^+^]_ex_ was restored to the chamber.

Because the [Na^+^]_in_ response time is long (29 s) compared with the perfusion time (3 s), the SMF dropped first and then increased to the steady state when the medium changes to lower [Na^+^]_ex_ (Figure 5A, red line). There is an inverse response for the perfusion experiment to the higher [Na^+^]_ex_ (Figure 5A). Therefore, it seems the BFM speed drop is reasonable. However, the number of stator units is also with a sudden change when the medium changes to lower [Na^+^]_ex_ (Figure 5A, green line). The number of stator units was approximately 10 in the 85 mM [Na^+^]_ex_ and drop to 5 right after 1 mM [Na^+^]_ex_ was applied. The number of stator units resurrected to 10 in approximately 2 min (Figure 5A). The phenomenon was examined in high temporal resolution data of a single motor as well (Figures 3B,D). Clearly, there was a fast stator number reduction and stepwise resurrection during the recovery part in the step-down [Na^+^]_ex_ (Figure 3D, inset).

We further examined BFM speed in different levels of [Na^+^]_ex_ changes (Figure 5B and Supplementary Figure 4) and the number of stator units dynamics (Figure 5C). Overall, the speed profiles are similar to the 85-01-85 mM perfusion experiments. Under step-down perfusion, the BFM speeds drop and gradually raise to the steady-state speed.

For 85-30 (mM), 30-05 (mM), and 85-05 (mM) perfusion experiments, the BFM speeds were approximately the same, and the motors recruited more stator units (Figures 5B,C). To verify the increase in stator number, we examined the speed data with high time resolution of a single motor (Supplementary Figure 5). The stepwise increase in speed is the evidence for recruiting new stator units. For 30-85 (mM), 05-30 (mM), and 05-85 (mM) perfusion experiments, the BFM speed remains approximately the same level, and the number of stator units reduced (Figures 5B,C).

For 85-01 (mM) and 30-01 (mM) perfusion experiments, the stator units reduced first and then resurrected. This is different from the reversible Hill–Langmuir model. The two-stage transition may be due to the cell’s stator response or physiological response (membrane potential). We examined the perfusion experiment 85-00-85 (mM) in the wild-type *E. coli* proton-driven motor (Supplementary Figure 6). When sodium was completely removed, the wild-type motor maintains the same speed. The result suggests that the cell physiology and membrane potential remain the same in the sodium perfusion experiments. It is likely the stator unit has extra mechanism of the sodium response.

To further quantify the initial stator unit jump in the step-down [Na^+^]_ex_ perfusion experiment, we compiled the statistic of the number of stator unit changes (Figure 5D). For 85-05 (mM) and 30-05 (mM) perfusion, the number of stator units in average shifts approximately 1 and 2, respectively. The positive numbers reflect the increasing affinity for higher stator numbers to maintain the power output. For 85-01 (mM), the stator unit dropped at least 5 of 10 stator units on average, implying that the motor may encounter quick remodeling of stator units.

To characterize the response time scale of the stator unit dynamics, the stator unit resurrection traces are fitting with Eq. 6. In Figure 5E, one can find that these *k*_on_ and *k*_off_ are very different from stator unit resurrection from 00-01 mM perfusion experiments. The *k*_on_ for 85-01 mM perfusion is approximately 0.03 (1/s), which is four times faster than 00-01 mM perfusion. These results may imply that the stator recruitment process may be different in these two conditions.

## Discussion

In this report, we applied computer-controlled microfluidic devices for fast perfusion to investigate the stator dynamics. The high-throughput and systematic data collection provides a nonbias and ideal data set for analysis.

We found that although the chimeric BFM has constant speed over a wide range of sodium ion concentrations, the stator occupancy does not saturate to its maximal capacity. Previous studies have shown that contribution of a stator unit to the motor speed increases with the rise in sodium concentration, Eq. 2 (Lo et al., 2006). Thus, the plateau region of the speed, in compensation, is the reduction of functional number of stator units on the motor as the SMF increases. This is against the long-held belief that stator unit’s stability is proportional to the sodium ion concentration. Wadhwa et al. (2019) have reported the BFM stator stability decreases when the external load decreases and rotational speed increases. Although the molecular mechanism remains unclear, our results show the same conclusion that the BFM stator is less stable in the high-speed conditions. As the output power of a motor is proportional to the rotational speed, the wide-range constant speed suggests a wide range of constant power output, whereas the SMF varies. The wide range of BFM speed may provide biological advantage for bacterial cells to maintain chemotaxis and optimize the energy usage in different external environments.

The stator binding and unbinding rate *k*_on_ and *k*_off_ have different sodium dependencies. The stator *k*_on_ is a constant for the [Na^+^]_ex_ higher than 5 mM. This suggests that it required at least 5 mM [Na^+^]_ex_ to trigger a stable activation. On the other hand, the unbind rate *k*_off_ is lowest at 5 mM [Na^+^]_ex_ and increases for both increasing and decreasing [Na^+^]_ex_. For higher [Na^+^]_ex_ end, the BFM drives faster, and the ion flux increases. Similar to the low-load high-speed condition, the stator *k*_off_ increases (Nord et al., 2017a). A recent stator structure report shows an ion reservoir region in the cytoplasmic region of stator units (Santiveri et al., 2020). Therefore, the ion discharge step might be a rate-limiting step for stator to work at high [Na^+^]_ex_ and high-speed region. However, how the rate-limiting step destabilizes the stator binding remains unclear. For low [Na^+^]_ex_ end, the number of stator units is reduced due to the low binding affinity. Our finding is consistent with the model that stator stability is mediated by the external load, IMF, and other factors that would affect the force generating process (Armitage and Berry, 2020).

Sodium-driven stators in *V. alginolyticus* showed the [Na^+^]_ex_–dependent stator polar localization (Fukuoka et al., 2009). However, there are reports demonstrating that the loss of IMF does not affect the assembly of the stator units in *Salmonella* and *E. coli* (Morimoto et al., 2010; Suzuki et al., 2019). The chimeric stator unit, PotB7^E^, is composed of *E. coli* cell wall binding domain that is critical for the stator assembly to the motor, as shown in Figures 1A,C. In our experiment of 85-00 mM perfusion, the BFMs stopped immediately. In the current stator unit recruitment model, the stator units can have only bound and unbound states. One might imagine a possible condition that a stator unit could disengage with the rotor but not lose the cell wall binding, leading to a potential mechanism for the IMF independent stator assembly in *E. coli* (Morimoto et al., 2010). To verify this possibility, we conducted a single-cell fluorescent measurement of the eGFP-fused stator units, Supplementary Figure 7A. In a tethered cell setup, the fluorescence intensity indicated that the number of stator units reduced immediately after the 85-00 mM perfusion, Supplementary Figure 7B. The fluorescence also recovered after the 85 mM [Na^+^]_ex_ was restored. The BFM speed is consistent with stator fluorescence signal, Supplementary Figure 7B. We can confirm that the stator units disassembled from the rotor in the 0 mM [Na^+^]_ex_ conditions, showing the stator assembly and disassembly were sensitive to SMF in this chimeric motor. The data also support the model that bounded stator units can physically leave the rotor region, and the diffusing stator units can reassemble to the motor.

How the sodium ion affects the stator assembly remains unclear. Recently, Terahara et al. reported that OmpA-like domain folding is sodium-dependent in the sodium-driven MotPS stator unit (Terahara et al., 2017). However, the OmpA-like domain in the chimeric PotB7^E^ is from proton-driven *E. coli* MotB. We speculate that the ion transition through the stator units is the key for stator assembly in this chimeric motor as previously reported for *V. alginolyticus* (Fukuoka et al., 2009).

**FIGURE 6:**
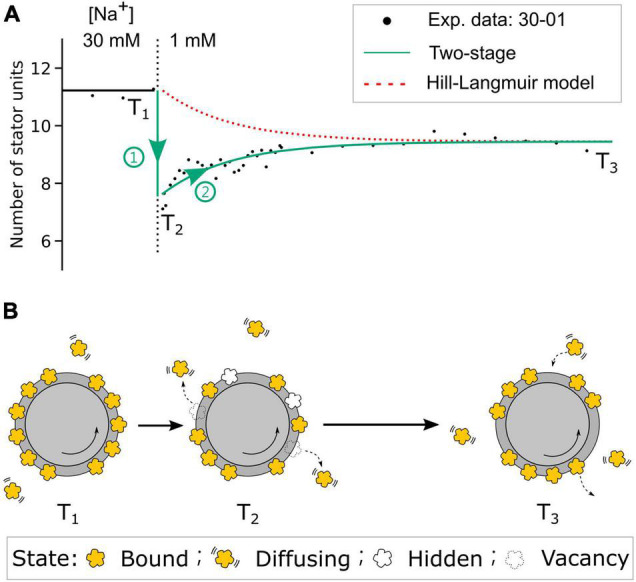
The two-stage stator unit response model. **(A)** The comparison of stator unit numbers dynamics in the step-down 30-01 mM [Na]_ex_ experiment from the two-stage model and the Hill–Langmuir absorption model. Black dots are the experimental data of stator unit numbers. The red dotted line represents the expected stator unit number transition from the Hill–Langmuir absorption model. The green line represents the time course of stator unit number in the two-stage model. T_1_ and T_3_ are the time points of motors at [Na]_ex_ = 30- and 1-mM steady state, respectively. T_2_ is the time point right after the [Na]_ex_ step-down. **(B)** Model diagram of the stator states at three time points. At time point T_1_, the number of stator units is dynamically balanced on the motor. Right after the [Na]_ex_ step-down, stage 1 (green circle 1), the numbers of stator units reduced partially by stator units unbound and partially by stator units enter the bound-but-not-functioning hidden state. In the second stage (green circle 2), the stator units resurrect to the steady state until the motor reaches the new steady state at time point T_3_. As the new dynamic balance is formed, the average number of bound stator units is less than that at T_1_.

From the steady-state BFM speed and stator unit number data (Figures 2B,C), we expect no stator change in the 85-01 (mM) perfusion. Surprisingly, the stator unit number initially dropped and then resurrected to stator number in the steady state. The resurrection process is faster than these in 00-01 and 30-01 (mM) perfusion. But we did not find the reverse process as 01-85 mM perfusion experiments. In order to gain a deeper insight into the resurrection after overshooting in step-down sodium perfusion, we conducted fluorescence measurement of eGFP-fused stator units in tethered cell setup. For 30-00 and 30-01 mM perfusion experiments, as expected, tethered cell rotational speed reduced to zero and maintained the same respectively, Supplementary Figure 7C. However, the fluorescence intensity of the motor region drops right after the perfusion indicates a stator unit disassembled process, Supplementary Figure 7D. The stator unit dropping less in 30-01 mM perfusion suggests there are remaining stator units still bound to the BFM and maintaining the speed. But the resurrection is faster than 00-01 mM perfusion (Figure 5E). Based on these data, we proposed a two-stage stator unit response model for [Na^+^]_ex_ step-down, as shown in Figure 6. Right after the [Na^+^]_ex_ step-down, some stator units were disassembled, and some were still in the bind but not in the functional state. Therefore, the number of functional stator units shows a rapidly excessive reduction and then a second-stage resurrection to the steady state. A recent report using kinetic analysis on BFM speed showed multiexponential shapes of dwell-time distribution. It suggested the existence of a hidden state in the stator unit exchange process (Shi et al., 2019). The bound but not functioning state is one of the possible states of a short-life hidden state.

In light of the discussion, we can have three conclusions. First, the chimeric stator is less stable in the higher sodium concentration. Second, the stator assembly and dissemble are sodium dependent. Third, there is a possible stator bound but no functioning state existed. On the basis of our findings and available information, the reversible Hill–Langmuir model is too simple to describe the stator unit dynamics. Correlated microscopy combining BFM speed and fluorescence measurements with computer-controlled microfluidic devices could provide further insight into the stator unit dynamics. It is important to study the protein exchange in response to various factors such as load, temperature, and energetics to obtain a complete understanding of BFM working mechanisms.

## Data Availability Statement

The raw data supporting the conclusions of this article will be made available by the authors, without undue reservation.

## Author Contributions

T-SL and C-JL designed the project. SK, HF, AI, and MH constructed strains and design the experiments. T-SL conducted the experiments, analyzed the data, and wrote the manuscript. C-JL analyzed the data and wrote the manuscript. All authors contributed to the article and approved the submitted version.

## Conflict of Interest

The authors declare that the research was conducted in the absence of any commercial or financial relationships that could be construed as a potential conflict of interest.

## Publisher’s Note

All claims expressed in this article are solely those of the authors and do not necessarily represent those of their affiliated organizations, or those of the publisher, the editors and the reviewers. Any product that may be evaluated in this article, or claim that may be made by its manufacturer, is not guaranteed or endorsed by the publisher.

## Abbreviations

BFM: bacterial flagellar motor
IMF: ion-motive force
SMF: sodium-motive force
BFI: back focal-plan interferometry
[Na^+^]_ex_: extracellular sodium ion concentration
[Na^+^]_in_: internal sodium ion concentration
*V*_m_: membrane potential.
