# Supplementary material for: Stator Dynamics Depending on Sodium Concentration in Sodium-Driven Bacterial Flagellar Motors
Source: Front Microbiol. 2021 Nov 26;12:765739. doi: 10.3389/fmicb.2021.765739 (PMC8661058; doi:10.3389/fmicb.2021.765739)
Supplement: Supplementary file 1 [file Presentation_1.pdf]

Stator dynamics depending on sodium concentration in sodium-driven bacterial flagellar motors

Supplementary Material

Tsai-Shun Lin[1], Seiji Kojima[2], Hajime Fukuoka[3], Akihiko Ishjima[3], Michio Homma[2] and Chien-Jung Lo[1*]

[1]Department of Physics and Center for Complex Systems, National Central University, Taoyuan City, Taiwan 32001, Republic of China

[2]Division of Biological Science, Graduate School of Science, Nagoya University, Chikusa-ku, Nagoya, 464-8602, Japan

[3]Graduate School of Frontier Biosciences, Osaka University, 1-3 Yamadaoka, Suita, Osaka 565-0871, Japan

***Correspondence:**
Corresponding Author
cjlo@phy.ncu.edu.tw

Supplementary Material

Supplementary text

Materials and Methods

1. Observation of stator unit with fluorescence microscopy
   Bacteria flagellar were fixed on anti-FliC antibody (Nishiyama et al., 2012) coated slides. The slides were prepared as follows. Coverslip and slide were pre-cleaned with ethanolic KOH and assembled as a flow chamber. The clean chamber was silanized with APTES (Cat. No: 440140, SIGMA) for 30 minutes and the residues were flush away with MB. Gold nanoparticles (Cat. No: 742031, Sigma-Aldrich) working as drifting correction markers were injected into the chamber for 5 minutes and flushed away with MB. The chamber surface was further activated with maleimide group through 5 mM $SM(PEG)_{24}$ (Cat. No: 22114, Thermo) for 3 hours and flushed with coupling buffer (10 mM potassium phosphate, 85 mM NaCl, 10 mM EDTA, pH 7.0). While waiting for the surface activation, FliC antibody were modified by adding sulfhydryl groups with 4 mg/ml Traut's reagent (Cat. No: 26101, Thermo) in coupling buffer (pH 8.0). The remaining reagent was removed by the desalting column (Cat. No: 89890, Zeba Spin) with the coupling buffer. Modified antibody was injected into the surface-activated chamber overnight at 4 degrees and flushed away with coupling buffer. The slides were preserved at 4 degrees until used. Before experiments, the chamber solution was replaced with MB possessing desired $[Na^+]_{ex}$. Harvested cells were injected into an anti-FliC antibody coated slide for 20 minutes. The none tethering cells were removed by MB.
   The fluorescent experiments were imaged by a Nikon Ti-U microscope equipped with a 100x objective (N.A. 1.49), a 2.5x relay, an EMCCD camera (Photometrics Evolve 512), and a 488 nm laser (Coherent Orbit) with a TIRF illumination system. The relative pixel size in an image is 64 nm. The laser power is around 48.3-59.5 W/cm^2. The motor's fluorescent intensity was estimated as the previous report (Fukuoka, 2017) with correction factor 1. Two overlaid regions of interest (R_1 and R_2) were centered at the fluorescent spot in the tether cell's rotational axis. The region size is 5*5 pixels for R_1 and 9*9 pixels for R_2. The fluorescent motor intensity is inside the R_1 area. The average intensity in the complement area of R_1 and R_2 is taken as the average background per pixel. The motor fluorescent intensity F_{motor} is calculated by removing background with the following equation:
   $$F_{motor} = I_1 - \frac{I_2 - I_1}{A_2 - A_1} \times A_1$$
   where I_1/I_2 is the total fluorescent intensities of R_1/R_2, and A_1/A_2 are total pixel number in R_1/R_2.
2. Supplementary Figures 2. The images were recorded as follows. Each tethered cell was recorded for 30 frames with 30 ms exposure time per frame. The BFM fluorescent intensity was derived by averaging the signals from 30 frames to reduce the fluctuation from rotating tethered cells.
3. Supplementary Figure 7B. The images were recorded as follows. These were three stages X-Y-X mM perfusion experiments with 20-20-80 min duration. Bright-field and fluorescence images were taken sequentially 10 frames and 1 frame per minute respectively. Each frame had an exposure time of 30 ms.
4. Supplementary Figure 7CD. The images were recorded as follows. These were two stages X-Y mM perfusion experiments with 3-3 min duration. In each stage, 10 frames of bright field images and 1 frame of a fluorescent motor image were taken per 10 seconds controlled by a program. Each frame had an exposure time of 30 ms. The fluorescent intensity and motor speed in each buffer were derived by averaging the results in 3 minutes (18 recordings).

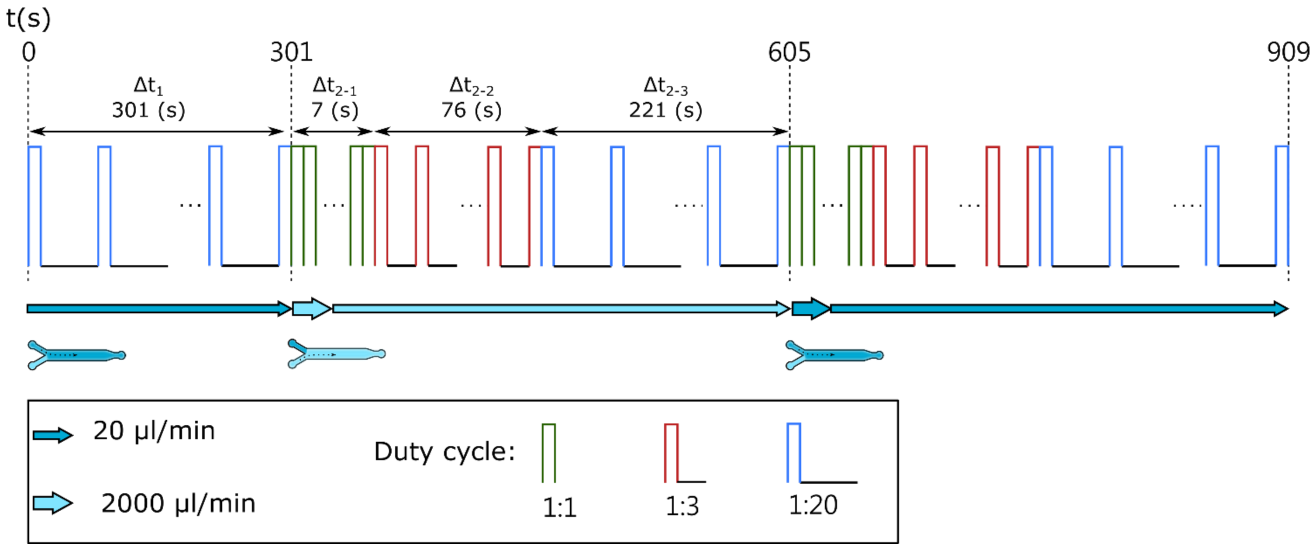

Supplementary Figure 1. Schematic of dynamic imaging intervals. For the first stage (Δt_1), about 5-minute period, the steady rotations were recorded one second in every 20 seconds durations (duty cycle 1:20). For the second stage, the recording sequence was continuous 7 seconds ($\Delta t_{2\text{-}1}$, duty cycle 1:1), one second in every 3 seconds ($\Delta t_{2\text{-}2}$, duty cycle 1:3) for total 76 seconds, and one second in every 20 seconds ($\Delta t_{2\text{-}3}$, duty cycle 1:20) for the rest 221seconds. Stage 3 is a repeat of stage 2.

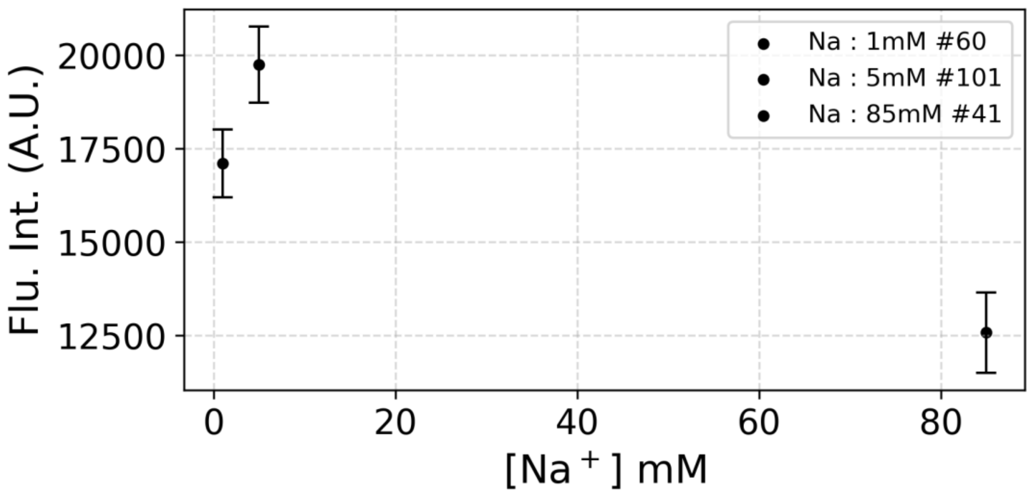

Supplementary Figure 2. The number of stator units bound to the rotor. The average intensity of rotating motors' fluorescent spot as a function of sodium concentration. There are more stator units in a BFM at 5 mM $[Na^+]_{ex}$ than those at 85 mM $[Na^+]_{ex}$. The error bar is the standard error of the mean. The number behind the hash key in the legend shows the number of sampled BFMs.

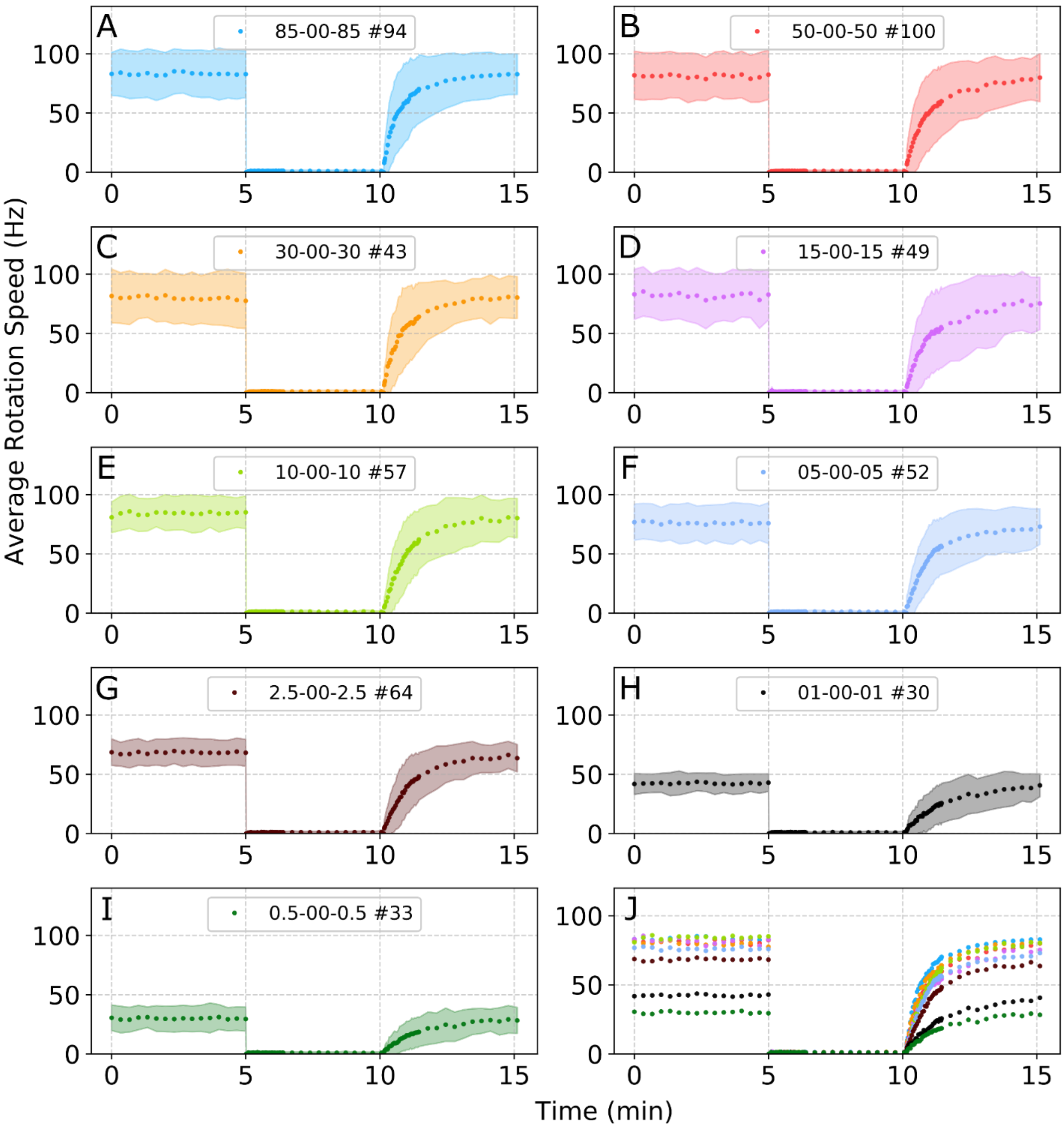

Supplementary Figure 3. (A-I) Temporal evolution of BFM rotational speed at various sodium perfusion experiments. The dots and the colored region represent the mean value and standard deviation respectively. The number of motors analyzed was indicated in the figures (#). (J) The summary of average BFM speed from (A-I). The line colors are consistent in (A-I).

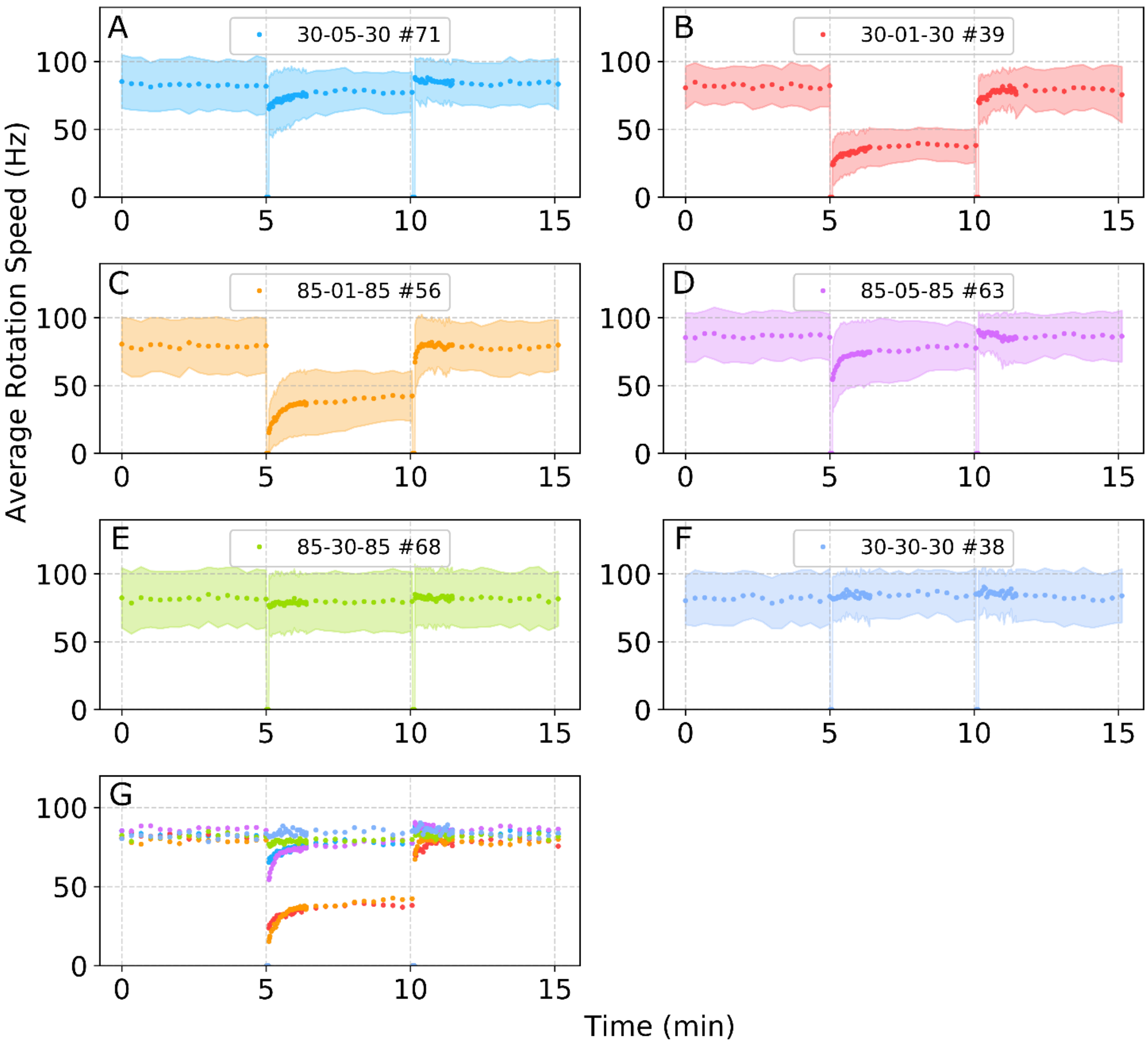

Supplementary Figure 4. (A-F) Temporal evolution of BFM rotational speed at various non-zero sodium perfusion experiments. The dots and the colored region represent the mean value and standard deviation respectively. The number of motors analyzed was indicated in the figures (#). (G) The summary of average BFM speed from (A-F). The line colors are consistent in (A-F).

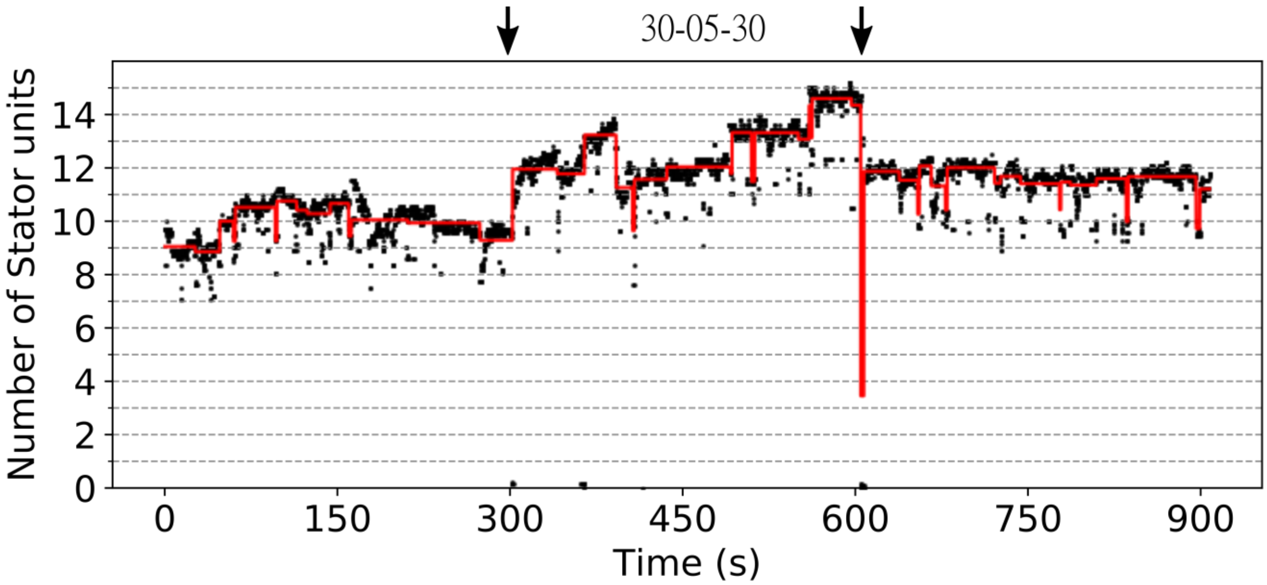

Supplementary Figure 5. High spatial temporal resolution recording in fast perfusion experiments 30-05-30. The arrows indicate the time $[Na^+]_{ex}$ perfused. There are stepwise speed increments after 5 mM $[Na^+]_{ex}$ introduced. The deriving of the step-wise red line is the same as in Figure 2.

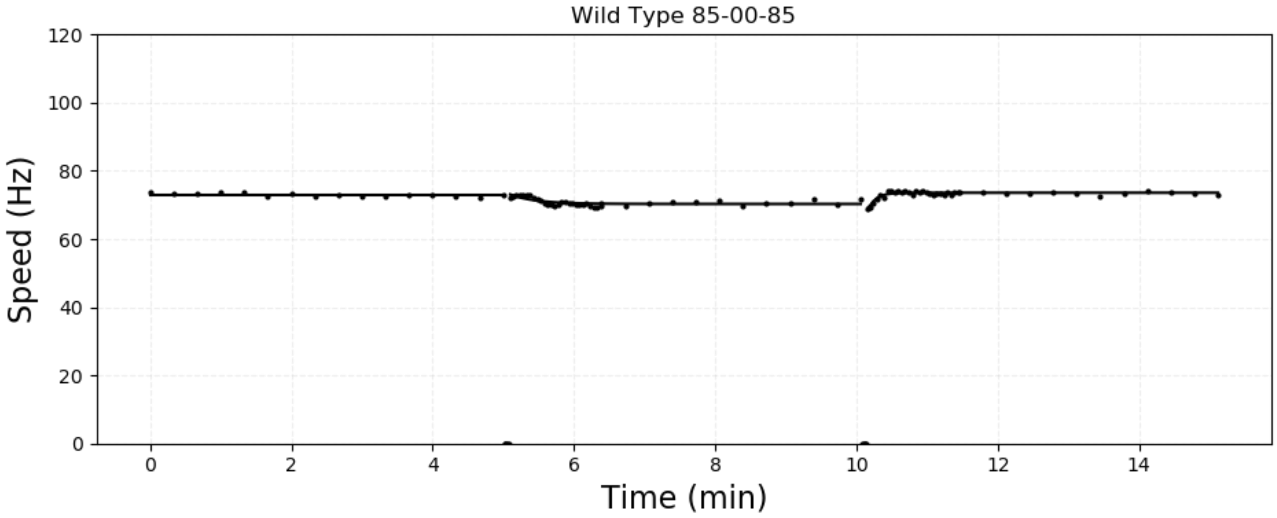

Supplementary Figure 6. Sodium perfusion experiments on wild-type *E. coli* proton-driven motor. Perfusion experiment 85-00-85 (mM) was applied in the wild-type *E. coli* proton-driven motor. When sodium was totally removed, the wild-type motor maintains the speed. The black lines are derived by fitting with a constant value for first stage and an exponential relaxation curve for the second and third stages.

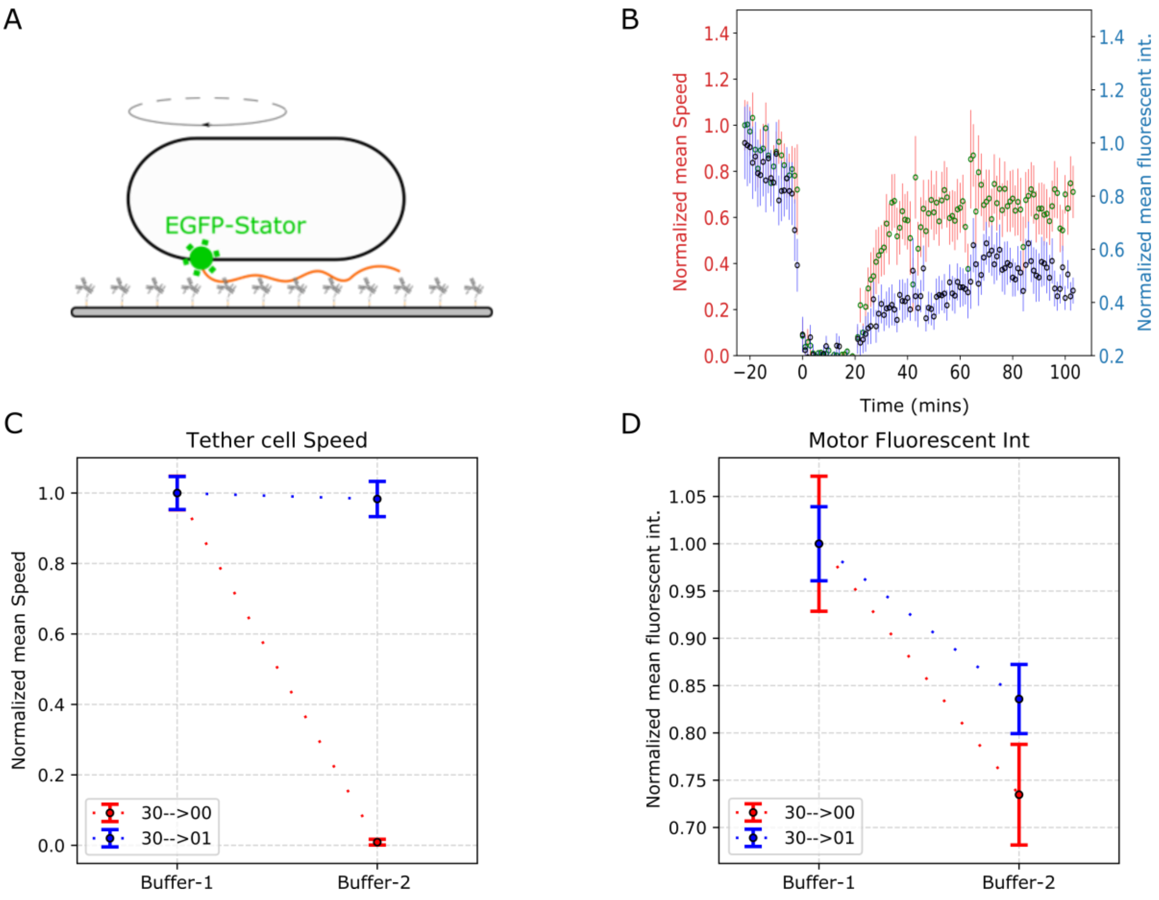

Supplementary Figure 7. Observation of stator unit dynamics with fluorescence microscopy (A) Schematic of experimental condition. Tethered cell experiments were conducted using bacterial flagellum immobilized to the FliC-antibody coated coverslip. The stator unit protein PomA is fused with eGFP and rotor protein FliN is fused with mCherry. The stator fluorescence signal of functional BFMs were recorded by TIRF microscopy under sodium perfusion experiments. (B) Temporal evolution of BFM speed and stator fluorescent intensity during the perfusion experiments. BFMs were in 85 mM $[Na^+]_{ex}$ from -20 to 0 min. At time 0, $[Na^+]_{ex}$ were changes to 0 mM and restored back to 85 mM $[Na^+]_{ex}$ at time 20 min. Motor fluorescent intensity and speed dropped to the lowest value after removing sodium and recovered after sodium was restored. The error bar indicates the standard error of the mean. The data were collected from 27 cells. (C) BFM speed prior to and right after the 30-00/30-01 mM perfusion. When the sodium ion was removed to 0 mM, the tethered cells stopped rotation immediately. However, the rotation kept in 1mM $[Na^+]_{ex}$ condition. (D) BFM speed prior to and right after the 30-00/30-01 mM perfusion. The stator fluorescent intensity dropped in both conditions. However, the intensity dropped more in 0 mM $[Na^+]_{ex}$. The error bar is the standard error of the mean. Data were collected from 6 cells for $[Na^+]_{ex}$ 30-00 mM perfusion and 10 cells for 30-01 mM perfusion.

Reference

Nishiyama, M. and Kojima, S. (2012). Bacterial motility measured by a miniature chamber for high-pressure microscopy. *Int. J. Mol. Sci.* 13, 9225–39. doi:10.3390/ijms13079225.

Fukuoka, H. (2017). "Direct Imaging of Intracellular Signaling Molecule Responsible for the Bacterial Chemotaxis,"in *The Bacterial flagellum*. 215–226. doi:10.1007/978-1-4939-6927-2_17.
